# Direct Differentiation of Human Embryonic Stem Cells to 3D Functional Hepatocyte-like Cells in Alginate Microencapsulation Sphere

**DOI:** 10.3390/cells11193134

**Published:** 2022-10-05

**Authors:** Xiaoling Xie, Xiaoling Zhou, Tingdang Liu, Zhiqian Zhong, Qi Zhou, Waqas Iqbal, Qingdong Xie, Chiju Wei, Xin Zhang, Thomas Ming Swi Chang, Pingnan Sun

**Affiliations:** 1 Stem Cell Research Center, Shantou University Medical College, Shantou 515041, China; 2 Guangdong Chaozhou Health Vocational College, Chaozhou 521000, China; 3 The Center for Reproductive Medicine, Shantou University Medical College, Shantou 515041, China; 4 Guangdong Provincial Key Laboratory of Infectious Diseases and Molecular Immunopathology, Shantou University Medical College, Shantou 515041, China; 5 Guangdong Provincial Key Laboratory of Marine Biotechnology, Institute of Marine Sciences, Shantou University, Shantou 515063, China; 6 Laboratory of Molecular Cardiology, First Affiliated Hospital of Shantou University Medical College, Shantou 515041, China; 7 Artificial Cells & Organs Research Centre, Departments of Physiology, Medicine & Biomedical Engineering, McGill University, Montreal, QC H3G 1Y6, Canada

**Keywords:** human embryonic stem cells, hepatocyte-like cells, 3D differentiation and culture, sodium alginate microspheres, artificial cells

## Abstract

Background: The lack of a stable source of hepatocytes is one of major limitations in hepatocyte transplantation and clinical applications of a bioartificial liver. Human embryonic stem cells (hESCs) with a high degree of self-renewal and totipotency are a potentially limitless source of a variety of cell lineages, including hepatocytes. Many techniques have been developed for effective differentiation of hESCs into functional hepatocyte-like cells. However, the application of hESC-derived hepatocyte-like cells (hESC-Heps) in the clinic has been constrained by the low yield of fully differentiated cells, small-scale culture, difficulties in harvesting, and immunologic graft rejection. To resolve these shortcomings, we developed a novel 3D differentiation system involving alginate-microencapsulated spheres to improve current hepatic differentiation, providing ready-to-use hESC-Heps. Methods: In this study, we used alginate microencapsulation technology to differentiate human embryonic stem cells into hepatocyte-like cells (hESC-Heps). Hepatic markers of hESC-Heps were examined by qPCR and Western blotting, and hepatic functions of hESC-Heps were evaluated by indocyanine-green uptake and release, and ammonia removal. Results: The maturity and hepatic functions of the hESC-Heps derived from this 3D system were better than those derived from 2D culture. Hepatocyte-enriched genes, such as HNF4α, AFP, and ALB, were expressed at higher levels in 3D hESC-Heps than in 2D hESC-Heps. 3D hESC-Heps could metabolize indocyanine green and had better capacity to scavenge ammonia. In addition, the 3D sodium alginate hydrogel microspheres could block viral entry into the microspheres, and thus protect hESC-Heps in 3D microspheres from viral infection. Conclusion: We developed a novel 3D differentiation system for differentiating hESCs into hepatocyte-like cells by using alginate microcapsules.

## 1. Introduction

In recent years, hepatocyte transplantation, as an effective treatment for acute liver failure and end-stage liver disease, has shown potential in clinical applications and has been widely studied in the field of liver regeneration [1]. Compared with liver transplantation, hepatocyte transplantation has the advantages of less trauma, low cost, repeatability, and long-term cryopreservation of transplantable cells [2]. However, the shortage of hepatocyte sources and immune rejection are the major limitations for hepatocyte transplantation in the clinic. The recent development of stem-cell-derived hepatocytes offers a potential solution for the lack of a transplant-cell source. Human embryonic stem cells (hESCs), derived from cell masses within the blastocyst, are immortal cells with the ability to differentiate into all somatic cell types, including hepatocyte-like cells, that can be widely used as an important tool for clinical and basic research [3]. Human embryonic stem-cell-derived hepatocyte-like cells (hESC-Heps) display great potential for in vitro studies of the development, function, and drug toxicity of the human liver (establishment of a human embryonic stem cell-based liver differentiation model for hepatotoxicity evaluations). To obtain hESC-Heps for clinical application, researchers continue to develop various methods to differentiate hESCs into hESC-Heps, such as using small molecules for differentiation to lower the cost [4,5], large-scale culture using a 3D suspension system or a rotating bioreactor [6,7], improving maturation by 3D-culture [8], and using microscaled, multilayered colonies to enhance homogeneity and maturation [9]. However, producing suitable hepatocytes for clinical applications remains challenging, especially for large-scale culture, development and retention of function, and production costs, as well as immunocompatibility. Thus, an appropriate, controlled, and scalable culture environment that leads to cell differentiation and function may alleviate the current practical limitations of stem-cell-derived hepatocytes.

Cell microencapsulation technologies are playing an increasingly important role in tissue engineering and regenerative medicine. Microcapsules are spherical micro-containers that use natural or synthetic polymer materials as a membrane wall shell to encapsulate drugs, bioactive substances, or living cells [10]. Microencapsulation can prevent cells, enzymes, and other biological macromolecules from escaping from the microcapsule, while small molecules and nutrients from the culture medium can freely enter and exit the semi-permeable membrane, so as to achieve large-scale cell culture and controlled//localized release of therapeutic factors [11]. Microencapsulation not only provides a good 3D microenvironment for the growth of cells to ensure the large-scale culture of cells in vitro, but also confers immune isolation and biocompatibility. The selective permeability of the microcapsule shell protects the graft against the host immune system, effectively avoiding immune rejection in the process of allotransplantation. Development of various techniques for cell microcapsule preparation can enable a broad application of microspheres for cell-based therapy [12].

Encapsulation of cells in microcapsules allows for high-density cell culture and exchange of nutrients and oxygen and for secretion through the microcapsule membrane while protecting the transplant cells from being targeted by host antibodies [13,14]^-^. Microcapsules can promote the growth, differentiation, maturation, and protein secretion of various cell types, including bone marrow mesenchymal stem cells, mouse ESCs, and hESCs [15,16,17,18]. Sodium alginate is one of most widely used biomaterials for microencapsulation and has been successfully applied to many cell types [19,20,21]. Encapsulation of hepatocyte precursors in alginate microspheres can improve hepatic differentiation. Pasqua et al. encapsulated HepaRG cells (precursors of hepatocyte-like cells) in 1.5% alginate microspheres and observed that the cells self-rearranged in aggregates and fully differentiated over time, showing a wide range of liver functions [22]. On the other hand, encapsulation can facilitate transplantation of functional hepatocyte-like cells. Zhang et al. transplanted umbilical-cord-blood-derived hepatocyte-like cells into the abdominal cavity of rats with acute liver failure and effectively alleviated the symptoms of experimental rats [23]. To date, there have been few reports on the differentiation of hESCs into hepatocyte-like cells in 3D sodium alginate microspheres. Therefore, we studied the feasibility of directional differentiation of hESC-Heps in vitro following encapsulation into 3D microspheres prepared with sodium alginate hydrogel and assessed the function and characteristics of the hESC-derived hepatic cells.

## 2. Materials and Methods

### 2.1. Two-Dimensional Cell Culture and Cell Differentiation

All cell cultures were incubated in a humidified 37 °C, 5% CO_2_ atmosphere. The human ES cell line hES03 (WiCell Research Institute, Inc., Madison, WI, USA) was maintained in an undifferentiated state in 6-well Matrigel (Corning)-coated plates (BIOFIL, Guangzhou, China) in mTesR medium (Stem Cell Technologies, Vancouver, BC, Canada) containing 100 U/mL penicillin/streptomycin (P/S) (Gibco, Waltham, MA, USA). Media was aspirated and replaced with fresh media daily. Cultures were split and passaged every 4–5 days, whereby cells were detached following incubation with 1 mL of Accutase (Stem Cell Technologies, Vancouver, BC, Canada) at 37 °C for 5 min, resulting in a single-cell suspension, then seeded in Matrigel-coated 24-well plates at a density of 1.5 × 10^5^ for continued growth. Only passages 10 through 22 were used for experiments. To induce differentiation, cells were incubated for 3 days in RPMI 1640 medium (Gibco, Waltham, MA, USA) plus 1× B27 (Gibco, Waltham, MA, USA), 100 U/mL P/S (Gibco, Waltham, MA, USA), supplemented with 100 ng/mL activin A (PeproTech, Suzhou, China) and 3 μM CHIR99021 (Sigma-Aldrich, Saint Louis, MO, USA). Following treatment with activin A and CHIR99021, the differentiated cells were cultured in Knockout DMEM (Gibco, Waltham, MA, USA) containing 20% Knockout serum replacement (Gibco, Waltham, MA, USA), 1% DMSO (Sigma-Aldrich, Saint Louis, MO, USA), 1× GlutaMAX (Gibco, Waltham, MA, USA), 1× non-essential amino acid (NEAA) solution (Gibco, Waltham, MA, USA), 100 µM 2-mercaptoethanol (Sigma-Aldrich, Saint Louis, MO, USA), and 100 U/mL P/S (Gibco, Waltham, MA, USA) for 5 days. Subsequently, the differentiated cells were incubated in HepatoZYME-SFM (HZM) medium (Gibco, Waltham, MA, USA) containing 10 µM hydrocortisone-21-hemisuccinate (Sigma-Aldrich, Saint Louis, MO, USA) and 100 U/mL P/S (Gibco, Waltham, MA, USA), supplemented with 10 ng/mL hepatocyte growth factor (HGF) and 20 ng/mL oncostatin M (OSM) (all from PeproTech, Suzhou, China) for another 10 days. Human embryonic kidney cells (HEK293T) and the Huh7 cell line were maintained in high-glucose DMEM (HyClone, China) containing 10% fetal bovine serum (Gibco, Waltham, MA, USA) and 100 U/mL P/S (Gibco, Waltham, MA, USA).

### 2.2. Alginate Encapsulation

The definitive endoderm cells were encapsulated in 2% (*w*/*v*) alginate (Sigma-Aldrich, Saint Louis, MO, USA). The encapsulation was performed using a system based on the syringe extrusion method [24]. Briefly, on Day 3 of cell differentiation, the adherent cells were digested with dispase, and a confluent aggregate of adherent cells was transferred to Knockout DMEM medium with the above added supplement (Knockout SR, GlutaMAX, NEAA,2-Mercaptoethanol, DMSO and P/S), then mixed with an equal amount of 4% (*w*/*v*) sodium alginate to form a 2% sodium alginate cell suspension of 3 × 10^6^ cells/mL. The mixture of cell aggregates in 2% purified sodium alginate was rapidly extruded through a 27 G needle injection syringe and the droplets were allowed to fall into a gelation bath (100 mM CaCl_2_ (Sangon Biotech, Shanghai, China) + 20 mM D-fructose (Sangon Biotech, Shanghai, China) + 1 × HEPES (Gibco, Waltham, MA, USA), pH 7.4). The droplets produced were allowed to settle for 10 min in the gelation bath to ensure gel formation, after which the microbeads were washed twice with 0.9% saline, and then resuspended in differentiation medium as described above to continue differentiation for 15 days according to the above differentiation method.

### 2.3. HE and PAS Staining

Cells in culture dishes were fixed in cold methanol, and glycogen storage was visualized by PAS staining (Solarbio, G1281, Beijing, China). Binucleated cells were observed by H & E staining using a kit (Beyotime, C0105S, Jiangsu, China) following the manufacturer’s instructions, and images were taken with a microscope.

### 2.4. Immunofluorescence Staining

Cells were fixed in 4% paraformaldehyde (PFA) for 15 min, then blocked with 5% BSA prepared in PBS for 1–2 h at RT on a shaker. The main primary antibodies included mouse anti-Oct4 (Abcam, Ab19587, Shanghai, China), goat anti-SOX17 (Abcam, Ab224637, Shanghai, China), mouse anti-α-fetoprotein (AFP) (Sigma-Aldrich, A8452, Saint Louis, MO, USA), mouse anti-HNF4α (Sigma-Aldrich, SAB1412164, Saint Louis, MO, USA), and mouse anti-albumin (ALB) (Sigma-Aldrich, A6684, Saint Louis, MO, USA), which were all used at a 1:200 dilution, and incubated with cells overnight at 4 °C. After overnight incubation, cells were washed three times with PBST. Alexa Fluor 488-conjugated secondary antibodies were added and incubated with cells for 2 h at room temperature. Finally, cell nuclei were stained with DAPI (Beyotime, Jiangsu, China) and samples were visualized by means of fluorescence microscopy (Zeiss).

### 2.5. RNA Isolation and qRT-PCR

Total RNA was isolated from the cultured cells (about 5 × 10^5^) using RNAiso Plus Reagent (TaKaRa, Dalian, China) and was reverse transcribed by a ReverTra Ace qPCR RT kit (TOYOBO, Shanghai, China) according to the manufacturer’s protocol. Polymerase chain reaction (PCR) was performed with SYBR Green Master Mix (Thermo Fisher Scientific, Waltham, MA, USA). To release cells from microspheres for RNA extraction, the encapsulating alginate was depolymerized in a 55 mM sodium citrate (Aladdin, Shanghai, China) solution for 30 min [21,25]. To release the cells from the microspheres for RNA extraction, the encapsulated cells were incubated in an appropriate volume of sodium citrate solution (55 mM) at 37 °C for 5 min, then gently pipetted up and down, using a 1 mL pipette tip, a few times until the alginate microspheres were completely depolymerized. The obtained cell suspension was centrifuged at 300× *g* for 3 min and the supernatant discarded. RNAiso Plus Reagent was then added to the cell pellet for RNA extraction. One μg of total RNA was reverse transcribed into cDNA, using an RT-PCR Kit (FSQ-101, TOYOBO, Shanghai, China), and qPCR was performed using 2× Power SYBR Green Master Mix and an ABI 7500 PCR machine, with GAPDH for normalization of input RNA. RT-qPCR data were analyzed by the 2^−ΔΔCT^ method. Primer sequences are listed in Supplementary Table S1.

### 2.6. Permeability of Microspheres

A 2% cell-free saline sodium alginate solution was prepared as described above and allowed to form alginate microspheres. After the alginate microspheres were formed, microspheres were transferred to a 0.05% polylysine solution and allowed to stand for 10 min, then washed once with 0.9% normal saline and then changed into a 0.2% sodium alginate solution for 4 min. Microspheres were then washed once with 0.9% normal saline, and then transferred into a 3% citrate solution for 6 min to liquefy the core alginate, and to form sodium alginate–polylysine–sodium alginate (APA) microspheres [24]. Microspheres were washed once with 0.9% normal saline, then transferred to a new 96-well plate and FITC-labeled dextran solution was added to assess the permeability of the microspheres. The entry and exit of FITC-labeled dextran in/out of the microspheres was observed under an inverted fluorescence microscope and the fluorescence of the remaining FITC-labeled dextran in solution was measured with a microplate analyzer. 

### 2.7. Assessment of Intracapsular Cell Viability

To observe cell viability, on Days 3, 8, 14, and 18 of differentiation, encapsulated cells were collected, and a calcein/PI cell activity and toxicity assay was performed following the manufacturer’s instructions (Beyotime, Jiangsu, China). Calcein only fluoresces in living cells, while propidium iodide (PI) cannot penetrate the cell membrane of living cells, and can only stain dead cells whose cell membrane integrity has been damaged. The encapsulated cells were visualized by means of confocal microscopy (Zeiss, LSM 880, Jena, Germany).

### 2.8. MTT Assay

To evaluate cell viability, MTT solution (Sigma-Aldrich, Saint Louis, MO, USA) was prepared as a 5 mg/mL stock solution in dd H_2_O, filtered (0.22 μm, Millipore), and kept for no more than two weeks in the dark at 4 °C. Next, MTT working solution (0.5 mg/mL) was prepared from a 10x MTT stock solution (5 mg/mL) according to the volume of medium required by the 96-well plate. The appropriate amount of MTT working solution was added to 200 μL MTT solution/1 × 10^5^ cells, and cells were further incubated at 37 °C for 5 h. The supernatant was carefully removed and 150 µL DMSO/1 × 10^5^ cells was added to dissolve the formazan crystals. The supernatant was transferred to a 96-well microplate and the optical density (OD), at 592 nm, of each well was measured using a microplate reader.

### 2.9. Western Blotting

About 3 × 10^6^ of differentiated cells were washed once with PBS and then lysed with radio-immune precipitation assay (RIPA) buffer supplemented with a protease inhibitor cocktail (all from Beyotime, Jiangsu, China) on ice. The cell lysates were centrifuged at 12,000× *g* for 15 min at 4 °C, and the supernatant was collected. Protein concentration was determined using a BCA Protein Assay Kit (Beyotime, Jiangsu, China). Electrophoresis was performed in a 10% BIS-Tris gel. Proteins were then transferred to a nitrocellulose membrane and incubated overnight at 4 °C with 1:1000 dilution of primary mouse anti-α-fetoprotein (AFP) (Sigma-Aldrich, A8452, Saint Louis, MO, USA), mouse anti-albumin (ALB) (Sigma-Aldrich, A6684, Saint Louis, MO, USA), and mouse anti-β-actin (Solarbio, K200058M, Beijing, China) followed by incubation with 1:1000 dilution secondary antibody for 2 h at room temperature, and then protein bands were visualized using an enhanced chemiluminescence system. Protein expression was normalized to β-actin. All experiments were conducted at least three times. Western blotting was analyzed using Image J software, and the results expressed as the mean ± SD of the target protein/β-actin gray value.

### 2.10. Albumin Secretion

Cell culture medium supernatants were collected on Day 18 after induced differentiation. Albumin secretion was quantified using an albumin assay kit (Nanjing Jiancheng Bioengineering Institute Co., Ltd., Nanjing, China). Analyses were performed in conformity with the manufacturer’s instructions.

### 2.11. Ammonia Clearance Assay

Ammonia concentration was measured with an ammonia assay kit (Nanjing Jiancheng Bioengineering Institute Co., Ltd., Nanjing, China). Microspheres were co-incubated with various concentrations of ammonium chloride on Day 18 and co-incubated with 35 μmol/L ammonium chloride solution on Days 14, 16, 18, 20, and 22 for 5 h. Then, supernatant was collected and the concentration difference of ammonium chloride before and after incubation was measured. A standard curve was generated with a solution containing different concentrations of ammonium chloride. Absorbance at 630 nm was obtained using a microplate reader (INFINITE M200 Pro, TECAN, Männedorf, Switzerland).

### 2.12. Indocyanine Green Assay

Indocyanine green (ICG, Sangon Biotech, Shanghai, China) was dissolved in DMSO at 1 g/mL to prepare the storage solution. Then, the solution was added to cells to obtain a total concentration of 1 mg/mL in differentiation medium. The microspheres were incubated, in the medium containing ICG, at 37 °C and 5% CO_2_ for 24 h, then washed three times with NaCl to remove the ICG, after which the microspheres were refed with fresh medium for 24 h. ICG entry into and release from the cells was obtained by photographing under a microscope.

### 2.13. Statistical Analysis

Metabolic activity was normalized by the quantity of metabolite produced or consumed/hours of incubation/million cells seeded. Data are expressed as the mean ± standard deviation of the primary data (SD). A Microsoft Excel database and GraphPad Prism software were used to record and analyze all data. An unpaired Student’s *t*-test was used for data analysis. Differences with a *p*-value of <0.05 were considered statistically significant.

## 3. Results

### 3.1. Enzymatic Detachment Affects Hepatic Characteristics of 2D hESC-Heps in The Hepatocyte Stage

**Figure 1:**
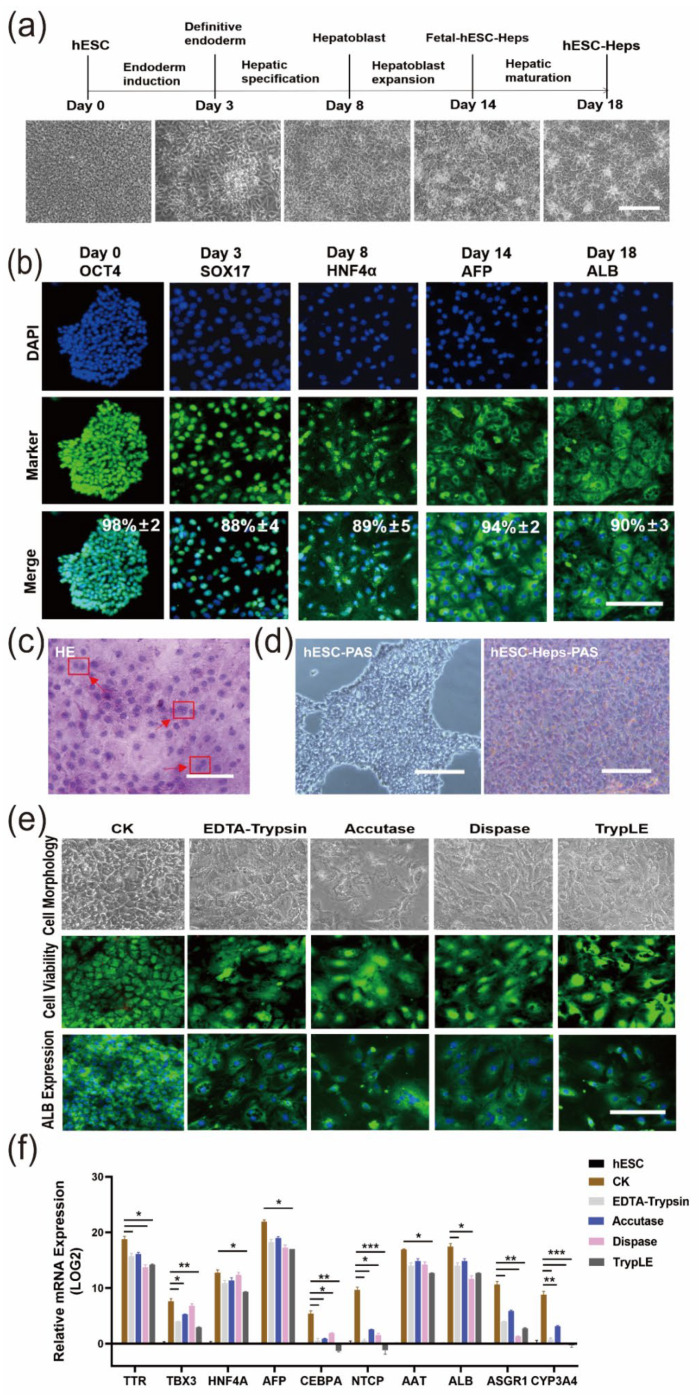
Enzyme digestion reduces the maturity of hESC-Heps differentiation. (**a**) Cell morphologies (bright field images) during differentiation of hESCs into hepatocyte-like cells in 2D monolayer culture (scale, 100 μm). (**b**) Expression of Oct4, SOX17, HNF4a, AFP, and ALB on Days 0, 3, 8, 14, and 18, imaged using fluorescence microscopy (*n* = 5 random regions) (scale, 50 μm). (**c**) HE stain showing double nuclei of differentiated hESC-Heps (scale, 100 μm). (**d**) Glycogen storage in hESC and hESC-Heps, as indicated by PAS staining (scale, 100 μm). (**e**) Effects of digestive enzymes on cell morphology, cell activity, and albumin expression after hESC-Hep digestion (scale, 100 μm). (**f**) Comparison of the mRNA expression levels of stage-specific genes in the digested and undigested hESC-Heps (scale, 100 μm). Data are the mean ± SD (*n* = 3) and analyzed by Student’s *t* test. * *p* < 0.05, ** *p* < 0.01, *** *p* < 0.001.

We developed a direct 2D differentiation approach for hESC-Heps (Figure 1a). The differentiation process consisted of four stages, including endoderm induction (Day 0–Day 3), hepatic specification (Day 3–Day 8), hepatoblast expansion (Day 8–Day 14), and hepatic maturation (Day 14–Day 18), generating endoderm cells, hepatic progenitor cells, and fetal and mature hepatocyte-like cells, respectively. The expression of protein markers in each stage was examined by immunofluorescence staining. OCT4 (Day 0), SOX17 (Day 3), HNF4α (Day 8), AFP (Day 14), and ALB (Day 18) were examined and highly expressed in the appropriate stage (Figure 1b). In addition, there were obvious binucleate cells, as shown by hematoxylin and eosin (H & E) staining, indicating that hESC-Heps exhibit the typical characteristics of hepatocytes (Figure 1c). hESC-Heps (Day 18) stained purple red after periodic acid-Schiff (PAS) staining, indicating glycogen accumulation (Figure 1d). In conclusion, the hESC-Heps in the hepatocyte stage displayed hepatocyte morphology and basic hepatic function. 

hESC-Heps normally are harvested by enzymatic detachment before application. In order to examine the influence of detachment enzymes on these cells, we digested hESC-Heps by using four different digestive enzymes (EDTA-trypsin, accutase, dispase, and TrypLE), and examined the change in morphology and viability of the cells, as well as albumin expression. As shown in Figure 1e, after digestion with various digestive enzymes and reseeding, hESC-Heps still maintained high cell viability, but the morphology significantly changed, showing an enlarged cytoplasm and decreased expression of albumin. In addition, we further detected the expression of hepatocyte-enriched genes, such as AFP, NTCP, ALB, and CYP3A4, and found that the expression of these specific genes decreased in the late stage of hESC-Hep differentiation after enzymatic digestion compared with that in the undigested cells (Figure 1f). These results suggest that enzymatic digestion may lead to dedifferentiation and affect the characteristics of hESC-Heps in a manner not conducive to further application.

### 3.2. Establishment of hESC-Hep Encapsulation Conditions

**Figure 2:**
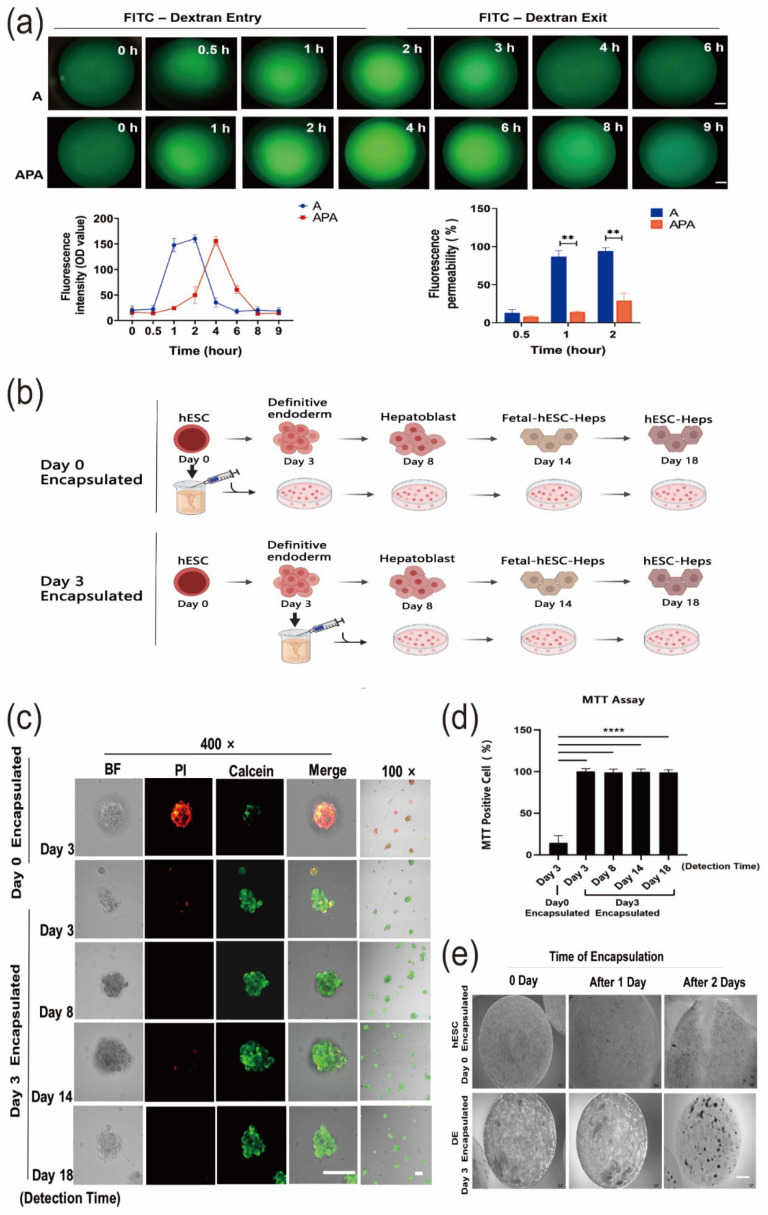
A novel 3D differentiation method using sodium alginate encapsulation. (**a**) Permeability/of sodium alginate microspheres and sodium alginate–polylysine–sodium alginate microspheres was detected by 60–76 kDa FITC-dextran (scale, 100 μm). (**b**) Schematic diagram of the 3D cell differentiation procedure. (**c**) Calcein/PI apoptosis staining assay for cell viability on Day 3 after Day 0 encapsulation and during the complete process of differentiation (Day 8, Day 14, and Day 18) after Day 3 encapsulation (scale, 100 μm). (**d**) MTT assay for detecting cell viability during the complete differentiation process following Day 0 encapsulation and Day 3 encapsulation. (**e**) Microsphere morphology following microsphere encapsulation on Day 0 and Day 3 (scale, 500 μm). Data are the mean ± SD (*n* = 3) and analyzed by Student’s *t* test. ** *p* < 0.01, **** *p* < 0.0001.

To encapsulate hESC-Heps, we optimized the encapsulation conditions, including encapsulation material and stage. hESCs were differentiated into hepatocyte-like cells by using small molecules and cytokines. To make sure that the small molecules and cytokines entered into the microspheres in time, we compared the permeability of microspheres embedded with sodium alginate (A) and sodium alginate–polylysine–sodium alginate (APA) by detecting entry and exit of 60–76 kDa fluorescein isothiocyanate (FITC)–dextran (Figure 2a). FITC–dextran could completely enter and exit out of the sodium alginate microspheres within 2 h, while it took more than 4 h for the FITC–dextran to completely enter and exit the APA microspheres, suggesting that the sodium alginate microspheres were permeable to small molecules and cytokines used in differentiation.

Another key condition that needed to be determined was the encapsulation stage of the cells. We encapsulated the cells at hESC stage Day 0 or Day 3 (definitive endoderm stage in 2D differentiation) and continued differentiating the cells into hepatocyte-like cells in the microspheres (Figure 2b). Calcein/PI fluorescence double staining was used to examine cell viability in the microspheres. For cells in microspheres encapsulated on Day 0 hESC stage, viability was decreased on Day 3 during hepatic differentiation as the percentage of cells stained with PI increased and the percentage of cells stained with calcein decreased. However, when encapsulated on Day 3 of differentiation, the cells retained high viability at all stages of differentiation (from Day 3 to Day 18) (Figure 2c). The MTT assay results also showed that percentage of living cells was only 14.58 ± 6.26% in the Day 0-encapsulated group compared to 99.36 ± 2.44% in the Day 3-encapsulated group after 3 days of differentiation. High cell viability (97.19 ± 2.62%) was still maintained in the Day 3-encapsulatedgroup when measured on Day 18 of differentiation. These results were also consistent with the results of calcein/PI double staining (Figure 2d). In addition, microspheres with cells encapsulated on Day 0 became larger with the increase in differentiation time, with many microspheres cracking after two days of differentiation. However, this did not happen to the microspheres with cells encapsulated on Day 3 (Figure 2e).

### 3.3. hESC-Heps in 3D Microspheres Display More Mature Hepatocyte Characteristics Than hESC-Heps in 2D Culture

**Figure 3:**
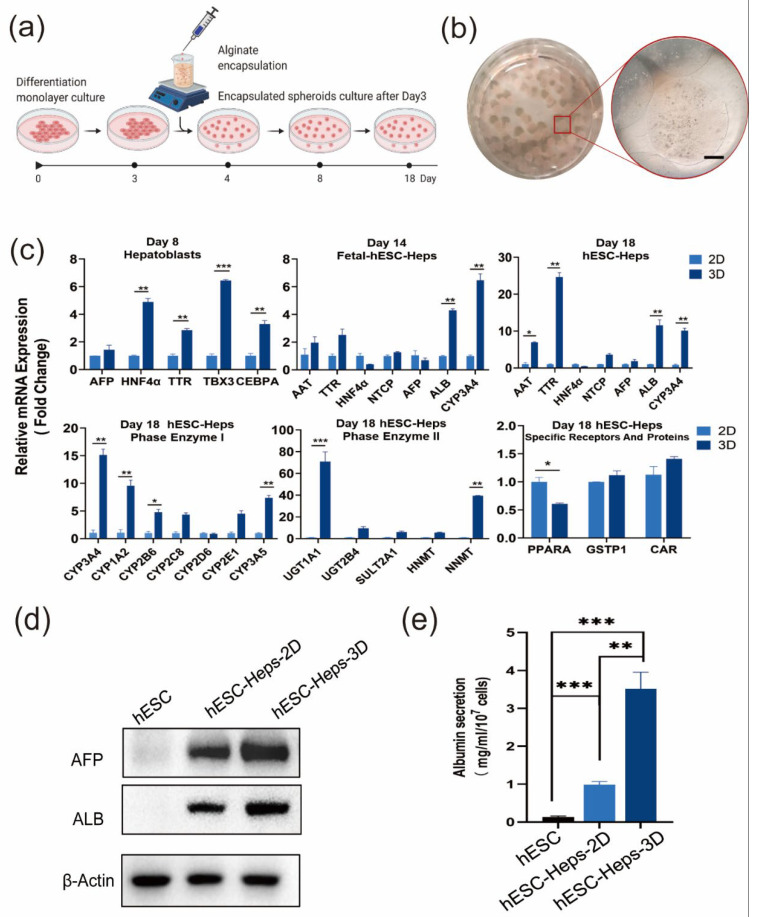
Differentiation and characteristics of hESC-Heps in 3D microsphere culture. (**a**) Schematic of the encapsulation and 3D cell-differentiation procedure. (**b**) Alginate microcapsules enclosing hESC-Heps (scale, 500 μm). (**c**) qRT-PCR for hepatocyte-specific markers on Days 8, 14, and 18, and expression of drug metabolizing enzymes and nuclear receptors of 3D hESC-Heps compared with 2D hESC-Heps. (**d**) Expression level of hepatocyte proteins in 3D hESC-Heps was compared with that of 2D hESC-Heps after induced differentiation for 18 days. (**e**) Albumin secretion of 2D hESC-Heps and hESC-Heps-3D was detected by a bromocresol green assay. Data are the mean ± SD (n = 3) and analyzed by Student’s *t* test. * *p* < 0.05, ** *p* < 0.01, *** *p* < 0.001.

The experimental setup designed for 3D differentiation is shown in Figure 1a. After 2D differentiation into definitive endoderm cells on Day 3, cells were mixed with sodium alginate solution and encapsulated using an ultra-small needle syringe and a calcium chloride solution. The encapsulated cells continued to differentiate into hESC-Heps through Day18 (Figure 3a). The sodium alginate microspheres appeared translucent under inverted phase-contrast microscopy, and the microsphere size ranged from 800 to 1600 μm diameter (Figure 3b). 

Real-time quantitative PCR results showed that compared with 2D hESC-Heps, 3D hESC-Heps highly expressed stage-specific genes (such as AFP and HNF4α on Day 8, and ALB, NTCP and CYP3A4 on Day 18), drug-metabolizing-enzyme genes (such as CYP3A4, CYP1A2, and UGT1A1) and nuclear receptor genes (such as GST and CAR), indicated that 3D hESC-Heps exhibited greater maturity than 2D hESC-Heps. This suggests that hepatocyte-like cells obtained by this 3D microsphere differentiation approach can be a potential resource for drug screening (Figure 3c). In addition, Western blotting showed that the expression levels of AFP and ALB in 3D hESC-Heps was higher than 2D hESC-Heps. Consistent results were obtained by detecting the albumin secreted in the cell supernatant, indicating that 3D microsphere culture can improve the ability of cell albumin secretion, which conforms to the requirements of hepatocyte therapy (Figure 3d,e).

### 3.4. Induction of Hepatocyte Functions in hESC-Heps and Antiviral Properties by 3D Microspheres

**Figure 4:**
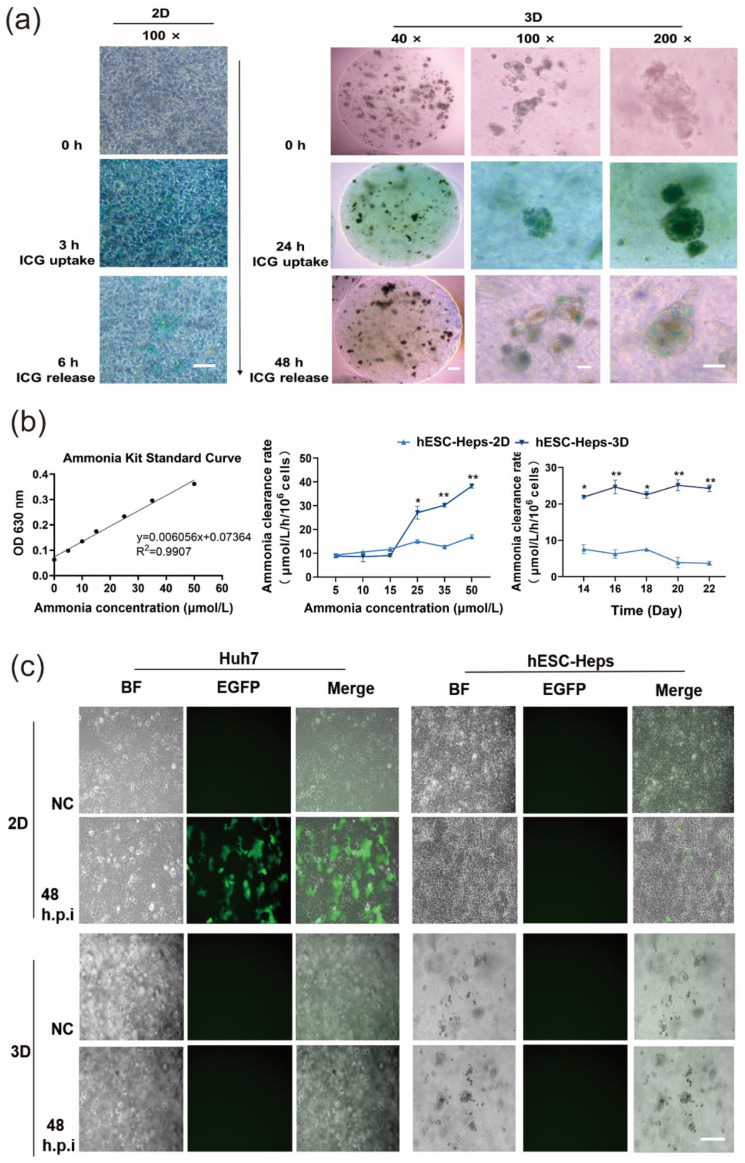
Characteristics and functionalities of hESC-Heps in 3D microsphere culture. (**a**) Indocyanine-green uptake and release by hESC-Heps. (**b**) Ammonia scavenging capacity of 2D hESC-Heps vs. 3D hESC-Heps. (**c**) Infection of Huh7 and hESC-Heps in microspheres following infection with VSVG-pseudotyped lentivirus encoding EGFP for 48 h. Infected cells show green fluorescence (NC, negative control). Data are the mean ± SD (n = 3) and analyzed by Student’s *t* test. * *p* < 0.05, ** *p* < 0.01. Scale, 100 μm.

Normal hepatocytes display excretion and metabolic transformation, and functional hepatocytes can absorb indocyanine green from the culture medium. 2D hESC-Heps successfully absorbed ICG after incubation with ICG-containing medium for 3 h, and 3D hESC-Heps could also absorb ICG after 24 h incubation (Figure 4a). This suggests that both 2D hESC-Heps and 3D hESC-Heps can metabolize ICG even though the rates of uptake and release ICG are different.

At the same time, we also examined the ability of the differentiated cells to remove ammonia. Blood ammonia in the human body ranges from 11 to 32 μmol/L [26,27,28]. Different concentrations of ammonium chloride solution were added to the co-culture with 3D hESC-Heps, and the concentration of ammonium remaining in the supernatant was determined after 5 h incubation. It could be seen that the ammonia-scavenging ability of 3D hESC-Heps was higher than that of 2D hESC-Heps at ammonia concentrations of 25, 35, and 50 μmol/L, respectively. We also further detected the ability of the cells to scavenge ammonia at a concentration of 35 μmol/L by measuring residual ammonia at different differentiation time points. The results showed that the scavenging ability of the cells remained at a relatively stable level after differentiation to a stage comparable to fetal hepatocytes (Figure 4b), indicating that 3D hESC-Heps can scavenge ammonia, and may have potential for the treatment of ammonia coma in liver failure. 

Alginate solution is known to have antiviral properties [29,30,31], so we examined whether sodium alginate hydrogels could protect microsphere-encapsulated hESC-Heps from virus infection. We infected cells with a VSV-G-pseudotyped HIV-1 (HIV-1(VSV))-lentivirus vector encoding an EGFP reporter gene and used Huh7 cells as the positive control, the results showed that both Huh7 cultured in 2D and 2D hESC-Heps could be infected, while neither Huh7 cultured in 3D nor 3D hESC-Heps could be infected (Figure 4c). This suggests that sodium alginate hydrogels can protect cells from infection. 

## 4. Discussion

Current differentiation platforms of stem-cell-derived hepatocytes are mainly constrained by small-scale culture, immaturity, and immune rejection. To solve these problems, the present study evaluated the feasibility of using microcapsules to construct a scalable tissue-culture environment to promote the differentiation of embryonic stem cells into hepatocytes.

We directly differentiated hESCs into hepatocyte-like cells in vitro and obtained functional hESC-Heps after differentiation for 18 days by using a stepwise method. Use of enzyme digestion for harvesting differentiated, Day 18 hESC-Heps caused dedifferentiation. The expression of liver genes was also affected by enzyme digestion (Figure 1). Therefore, we decided to encapsulate primary hESCs or hESC-derived cells at the early stage of the differentiation process and developed a system for direct differentiation of human embryonic stem cells into hepatocyte-like cells by using 3D sodium alginate hydrogel microspheres.

There are different forms of encapsulating materials, such as sodium alginate, sodium alginate–polylysine–sodium alginate (APA), and sodium alginate–chitosan–sodium alginate (ACA) in which polylysine is replaced with chitosan [32]. In addition to sodium alginate, the polymeric materials used in the preparation of cell microcapsules can also involve collagen [25,33], polyethylene glycol hydrogel [34,35,36,37], and other synthetic materials [38]. Alginate-based biomaterials have been shown to display very low or negligible toxicity [39]. Sodium alginate is a water-soluble aldehyde salt extracted from natural brown algae, and is a commonly used material for cell microcapsule and cell transplantation [40,41,42,43,44,45]. The Chang lab has previously studied alginate encapsulation of stem cells, and found syngeneic bioencapsulated bone marrow cells could increase survival of hepatectomized rats, by 90% through transdifferentiation into hepatocyte-like cells in the peritoneal cavity [46,47]. Here, we show sodium alginate-encapsulated hESC-derived endoderm cells can complete hepatic differentiation in fluorescent dextran-permeable 3D microspheres. Thus, we adopted sodium alginate encapsulation, which had better permeability than that of APA. 

To evaluate the feasibility of maintaining differentiation in a 3D microenvironment with sodium alginate, we encapsulated cell aggregates of hESCs, or hESC-differentiated definitive endoderm, into microspheres and determined cell viability by calcein/PI staining and MTT assay (Figure 2c,d). Unexpectedly, cells encapsulated at Day 0 of differentiation (hESC stage) displayed very low cell survival after differentiation for 3 days, whereas cells encapsulated at Day 3 (definitive endoderm stage) had much higher survival. Therefore, we encapsulated cells at the definitive endoderm stage for further differentiation in 3D microspheres. Our results showed that the alginate microenvironment can maintain a cell viability of more than 97% during the entire differentiation process (from Day 3 definitive endoderm to Day 18 hepatocyte-like cell stage), indicating that alginate microspheres can support cell differentiation and long-term culture. We encapsulated stem-cell-derived endoderm cells in aggregated form into microspheres, so that the cells could proliferate, interact with each other, and complete the process of differentiation. Previously it was shown that stem-cell-derived hepatocyte-like cells generated from 2D-differentiation systems have a phenotype closer to fetal than adult hepatocytes [48], whereas 3D culture systems have been shown to have stem-cell-derived hepatocyte-like cells of increased maturity [49]. Under 3D conditions, cell aggregation is liable to increase the hepatic niches and interactions between cells, thus further enhancing the function of hepatocytes. We characterized liver-specific gene expression to assess the maturity of differentiation of 2D hESC-Heps vs. 3D hESC-Heps at each stage during differentiation. The results showed that liver-enriched genes in 3D hESC-Heps are more highly expressed than those in 2D hESC-Heps. We further showed that 3D hESC-Heps can be recovered from depolymerized microspheres for downstream analysis and applications. 

However, improvements are still needed for high purity and full maturity of hESC-Heps generated from our current 3D hepatic differentiation system. For example, modulation of the Wnt signaling pathway during hepatic differentiation may be helpful in approaching to the above aims. As we know, Wnt modulation is crucial in directing the differentiation of hepatoblasts into hepatocytes or cholangiocytes by inhibition or activation in hepatogenesis [50]. Recently, two Wnt inhibitors, WIF-1 and DKK-1, were used to mass-produce human hepatocyte-like cells from embryoid bodies of hiPSCs [51]. WNT7B and WNT8B secreted from hepatocytes and cholangiocytes were identified to play important roles in achieving zone-specific characteristics, such as the enhancement of glutamine secretion and the citric acid cycle, and cytochrome P450 (CYP) 1A2 metabolism [52]. In addition, exclusion of R-spondin (a Wnt agonist) and FGF10 in culture medium enhanced self-renewal of iPSC-derived organoids with mature hepatic characteristics over long-term culture [53]. Another way to improve hESC-Hep differentiation is to co-culture hepatocyte-like cells with stromal cells [54,55]. Recently, the Ma lab encapsulated hiPSC-derived hepatocyte-like cells with stromal cells in biocompatible hydrogel capsules and successfully engrafted them in immunocompetent mice [54]. 

In addition to the solid alginate microspheres used in our study, other culture systems have been developed for 3D culture of hESC- or hiPSC-derived hepatocytes, such as alginate hollow microcapsules, alginate hollow fibers, and bioprinting [56,57,58]. One of the advantages of hollow spheres is that they provide a microenvironment for encapsulated cells with less restriction of their interactions, whereas, for a given volume, microcapsules have a much larger surface to volume relationship for solute transport when compared to hollow fibers. We will compare the efficiencies and qualities of hepatic differentiation among these systems in our future work. 

Transplantation of allogeneic or xenogeneic hepatocytes encapsulated in alginate microcapsules is a powerful therapeutic option to support liver recovery in the host, and has been verified in various animal models [59]. However, allogeneic cell microcapsules are associated with certain risks of viral infection during cell transplantation. Such viral infection during transplantation may have an adverse impact on transplantation. Alginate biomaterials have wide antiviral activities against nearly 17 types of viruses, including human immunodeficiency virus type 1 and hepatitis A, B, and C viruses [39]. In our study, 3D hESC-Heps in alginate microspheres were also protected from infection by a VSV-G-pseudotyped lentiviral vector (Figure 4), as well as human cytomegalovirus (HCMV). The antiviral mechanisms of alginate-based biomaterials are still not fully clear, but major attributions include viral aggregation and viral inhibition through interaction of alginate-based materials with components of the viral envelope [39]. Inhibition of viruses by ionic charge interactions has been reported, especially with sulfated polysaccharides of natural origin. Some researchers have studied the structure–activity relationship in the antiviral effects of seaweed polysaccharides and suggest that it may occur by inactivating the virus before exposure to the host cell [29,60]. Our results show that sodium alginate hydrogels insulate the virus from further infection of the cells inside the microspheres, which may be related to the interaction between the negative charge of the sodium alginate microcapsule and a viral-envelope component, which could block the interaction between the viral particle outside the microcapsule and its specific membrane receptors on the cell [29].

In summary, we established a novel method of in-situ differentiation of hepatocytes in 3D microspheres. Hepatocytes obtained by this method exhibit greater maturity and hepatocyte-like features than those obtained by traditional 2D differentiation and can further lead to large-scale culture to improve the in vitro production of functional hepatocyte-like cells for cell transplantation, drug metabolism testing, and biological artificial liver support.

## Data Availability

Not applicable.

## Supplementary Materials

The following supporting information can be downloaded at: https://www.mdpi.com/article/10.3390/cells11193134/s1, Table S1: The sequence of primers used for RT-qPCR.
