# Supplementary material for: Direct Differentiation of Human Embryonic Stem Cells to 3D Functional Hepatocyte-like Cells in Alginate Microencapsulation Sphere
Source: Cells. 2022 Oct 5;11(19):3134. doi: 10.3390/cells11193134 (PMC9562699; doi:10.3390/cells11193134)
Supplement: Supplementary file 1 [file cells-11-03134-s001.zip › cells-1832616-supplementary.pdf]

Supplemental Table S1. The sequence of primers used for RT-qPCR.

Gene	Primers	Product size（bp）
AFP	ACTGAATCCAGAACACTGCA<br>TGCAGTCAATGCATCTTTCA	174
HNF4α	ACATGGACATGGCCGACTAC<br>CGTTGAGGTTGGTGCCTTCT	123
TTR	AGAAAGGCTGCTGATGAC<br>GTGCCTTCCAGTAAGATTTG	145
TBX3	TTATGTCCCAGCGAGGGTGA<br>ACGTGGTGGTGGAGATCTTG	97
CEBPA	AGGAGGATGAAGCCAAGCAGCT<br>AGTGCGCGATCTGGAACTGCAG	141
AAT	AAATGAACTCACCCACGAT<br>ACCTTAGTGATGCCCAGT	141
NTCP	CATAGGGATCGTCCTCAAATCCA<br>GCCACACTGCACAAGAGAATG	90
ALB	CCCCAAGTGTCAACTCCA<br>GTTCAGGACCACGGATAG	129
ASGR1	CAGACCCTGAGACCCTGAGCAA<br>TCCTGCAGCTGGGAGTCTTTTCT	133
CYP3A4	AGATGCCTTTAGGTCCAATGGG<br>GCTGGAGATAGCAATGTTCGT	94
CYP1A2	CTGGGCACTTCGACCCTTAC<br>TCTCATCGCTACTCTCAGGGA	99
CYP2B6	TCTGGCCGGGGAAAAATCG<br>GGTCACAGAGAATCGCCGAAG	105
CYP2C8	GGAAAACGAATTTGTGCAGGAG<br>GTGGCAGAGAAACAATCCCTT	151
CYP2D6	CCAACGGTCTCTTGGACAAAG	79

	GGGTCGTCGTACTCGAAGC	
CYP2E1	GATGCCCTACATGGATGCTG<br>AAATGGTGTCTCGGGTTGCT	95
CYP3A5	GCAAACAGCCCAGCAAACA<br>GTCCATCGCCACTTTCCTTC	81
UGT1A1	TCCCACTTACTGCACAACAAG<br>GGTCCGTCAGCATGACATCA	73
UGT2B4	CAAATGTTGAGTTCGTTGGAGGA<br>CTGACGTGTTACTGACCATCG	138
SULT2A1	CTGGGAAAGACGTTAGAACCC<br>AAGTTGTGCTTTGTCCACTACAT	132
HNMT	GTGGAAAAAGTACGGATCACGC<br>GGCATTAAAGTTGCAGGTTTCAG	211
NNMT	GAGATCGTCGTCACTGACTACT<br>CACACACATAGGTCACCACTG	109
PPARA	TTCGCAATCCATCGGCGAG<br>CCACAGGATAAGTCACCGAGG	146
GSTP1	ACCATCCCTTTGGCTATTGAGA<br>TTCTGCCTGCGGAGTTTATCA	140
CAR	TGGGCACCATGTTTGAACAGT<br>GGGCAGGTCCTTAGTAAACTTG	167
GAPDH	ACAACTTTGGTATCGTGGAAGG<br>GCCATCACGCCACAGTTTC	101
